# Adenosine Deaminase Polymorphism Affects Sleep EEG Spectral Power in a Large Epidemiological Sample

**DOI:** 10.1371/journal.pone.0044154

**Published:** 2012-08-29

**Authors:** Diego Robles Mazzotti, Camila Guindalini, Altay Alves Lino de Souza, João Ricardo Sato, Rogério Santos-Silva, Lia Rita Azeredo Bittencourt, Sergio Tufik

**Affiliations:** 1 Departamento de Psicobiologia – Universidade Federal de São Paulo - São Paulo, Brazil; 2 Laboratório de Neurociências Clínicas (LiNC) – Departamento de Psiquiatria - Universidade Federal de São Paulo - São Paulo, Brazil; 3 Centro de Matemática, Computação e Cognição – Universidade Federal do ABC – Santo André, Brazil; University of Adelaide, Australia

## Abstract

Slow wave oscillations in the electroencephalogram (EEG) during sleep may reflect both sleep need and intensity, which are implied in homeostatic regulation. Adenosine is strongly implicated in sleep homeostasis, and a single nucleotide polymorphism in the adenosine deaminase gene (*ADA* G22A) has been associated with deeper and more efficient sleep. The present study verified the association between the *ADA* G22A polymorphism and changes in sleep EEG spectral power (from C3-A2, C4-A1, O1-A2, and O2-A1 derivations) in the Epidemiologic Sleep Study (EPISONO) sample from São Paulo, Brazil. Eight-hundred individuals were subjected to full-night polysomnography and *ADA* G22A genotyping. Spectral analysis of the EEG was carried out in all individuals using fast Fourier transformation of the signals from each EEG electrode. The genotype groups were compared in the whole sample and in a subsample of 120 individuals matched according to *ADA* genotype for age, gender, body mass index, caffeine intake status, presence of sleep disturbance, and sleep-disturbing medication. When compared with homozygous GG genotype carriers, A allele carriers showed higher delta spectral power in Stage 1 and Stages 3+4 of sleep, and increased theta spectral power in Stages 1, 2 and REM sleep. These changes were seen both in the whole sample and in the matched subset. The higher EEG spectral power indicates that the sleep of individuals carrying the A allele may be more intense. Therefore, this polymorphism may be an important source of variation in sleep homeostasis in humans, through modulation of specific components of the sleep EEG.

## Introduction

Sleep is a complex behavior that can be understood as an active process finely regulated and influenced by genetic and environmental factors [1]. The electroencephalogram (EEG) is a valuable tool for the characterization of brain wave oscillations seen across sleep stages. Studies have shown that individual EEG profiles in non-rapid eye movement (NREM) and REM sleep are genetically determined, being one of the most heritable traits in humans identified so far [2], [3], [4].

Sleep is thought to be controlled by two processes: circadian and homeostatic regulation [5]. A growing body of evidence suggests the participation of several neuromodulators in the homeostatic control of the sleep-wake cycle, with the adenosinergic system playing a relevant role [6].

Adenosine is a product of adenosine triphosphate metabolism, and is a signaling molecule described as a “sleep-promoting” substance. This neuromodulator is increased in the basal forebrain during prolonged wakefulness, and is highly correlated with slow wave activity in NREM sleep [7], [8], supporting its role in homeostatic control. The balance between adenosine production and degradation is controlled by an extensive network of enzymes, receptors, and transporters, which determines its intra- and extra-cellular concentration [6]. Adenosine deaminase (ADA) is an enzyme that irreversibly metabolizes adenosine to inosine, and may therefore modify the need for slow wave sleep during extended wakefulness [6], [9].

A common functional variant of the *ADA* gene has been described as a G to A transition (*ADA* G22A; rs73598374) which leads to the substitution of asparagine for aspartic acid at the eightieth codon of the gene [10]. It has been found that this functional polymorphism leads to a decrease in ADA activity in erythrocytes and lymphocytes [11], thus being a candidate variant in the regulation of adenosine-dependent sleep homeostasis. The *ADA* G22A polymorphism has been associated with higher sleep efficiency [12], longer slow wave sleep duration, and fewer reports of awakenings [13]. In addition, it has been suggested that its effects on sleep architecture may be dependent on caffeine–a powerful competitive antagonist of adenosine receptors [12].

Studies performed in relatively small samples and under strict experimental conditions have investigated the effect of this polymorphism on the sleep EEG. They reported increased activity in the delta frequency range in NREM and REM sleep [13], and in the recovery sleep after sleep deprivation [14]. The aim of the present study was to investigate the association between the *ADA* G22A polymorphism and sleep EEG spectral power in a large epidemiological sample from São Paulo, Brazil.

## Materials and Methods

### Ethics Statement

The study protocol was approved by the Research Ethics Committee of the Universidade Federal de São Paulo (CEP 0593/06) and all volunteers read and signed the informed consent form, according to the Declaration of Helsinki. This study is registered in ClinicalTrials.gov (Name: Epidemiology of sleep disturbances among adult population of the São Paulo City; Number: NCT00596713; URL: http://www.clinicaltrials.gov/ct2/show/NCT00596713?term=NCT00596713&rank=1).

### Studied Subjects

The initial sample consisted of 1042 participants in the São Paulo Epidemiologic Sleep Study (EPISONO)–a large population-based survey from São Paulo, Brazil. Details about the rationale, study design, and methodology have been published [15]. Socio-economic and demographic data, lifestyle factors, caffeine consumption habits, and health data were collected, as well as polysomnographic recordings and genetic data. From the initial sample, 958 individuals were genotyped for the *ADA* G22A polymorphism and for 31 ancestry informative markers to estimate the genetic ancestry proportions of the population, as previously described [12].

### Polysomnography and Clinical Assessment

A full-night polysomnography was performed using a digital system (EMBLA® S7000, Embla Systems, Inc., Broomfield, CO, USA) at the sleep laboratory. Physiological variables were monitored continuously, and recordings were scored according to standardized criteria [16], [17].

According to the International Classification of Sleep Disorders [18], obstructive sleep apnea syndrome (OSAS) was considered positive if individuals had an apnea-hypopnea index (AHI) between 5 and 14.9 and presented at least one of the following complaints: loud snoring, daytime sleepiness, fatigue, and breathing interruptions during sleep. Subjects with an AHI ≥15 were also considered positive, regardless of whether they had complaints. Participants were classified as insomniacs (DSM-IV) using both subjective and objective insomnia measures. Items extracted from the questionnaires were combined as an algorithm of the DSM-IV general criteria to define insomnia [19]. Individuals were evaluated for restless legs syndrome (RLS) based on standard diagnostic criteria [20], [21], [22].

### Spectral Analysis of Sleep EEG

A specific syntax in R (version 2.10.1) was used for spectral analysis of the sleep EEG. Waves from C3-A2, C4-A1, O1-A2, and O2-A1 derivations were decomposed into delta (<4 Hz), theta (4–7.9 Hz), alpha 1 (8–9.9 Hz), alpha 2 (10–12.9 Hz), beta 1 (13–17.9 Hz), beta 2 (18–29.9 Hz), and gamma (≥30 Hz) frequency bands using fast Fourier transformation, with sampling rate of 200 Hz, using epochs of 20 s. The filter settings used were according to standard criteria of sleep EEG data acquisition (low frequency filter of 0.3 Hz; high frequency filter of 35 Hz; time constant of 0.3 seconds and notch filter of 60 Hz). Artifacts were excluded as follows: the descriptive data (mean, standard deviation, median, and interquartile range) from each 20 s window were calculated, and the 5% of time windows with the highest signal amplitude (maximum–minimum) at each sleep stage were considered outliers and excluded from analysis. For validation of this procedure, an experienced polysomnographist subjectively identified and excluded artifacts in 30 polysomnographies. Results of the visual analysis and the R syntax were compared using a Kappa test (κ = 0.79; p = 0.002), showing good agreement between the two methods.

### Statistical Analysis

Z-score standardized values of the spectral power were used for analysis due to deviations from normality. The effects of the *ADA* G22A polymorphism on the spectral power at each sleep stage and at each electrode were tested using Student’s t-tests. After identification of frequency bands with statistically significant changes, GG and GA+AA genotype individuals were matched for age, gender, body mass index (BMI), European ancestry (estimated by the genetic ancestry informative markers), presence of sleep disturbance (OSAS, insomnia, or RLS), use of psychoactive medication which potentially affects sleep structure (phytotherapics, antidepressants, benzodiazepines, anticonvulsants, muscle relaxants, and zolpidem), and caffeine intake status (report of at least one cup of caffeine-containing drinks - coffee, tea, soft drinks or energy drinks) in the day prior to the polysomnography, using the *Genetic method* of the package *MatchIt*, implemented in R (version 2.14.0) [23]. This method determines the weight each covariate is given in order to find an optimal balance for matching and further select a subsample to be compared. A good matching relies on a propensity score, which, for the present data, is the covariate balance, or how much the groups to be matched have similar joint distribution of observed covariates [24]. Independent samples t-tests and chi-square tests were used to validate the matching efficiency. A weighted general linear model was applied to estimate the effect sizes (η^2^) and the observed power of the comparison of the significant bandwidths between *ADA* G22A genotype groups. Statistical analyses were performed using PASW Statistics 18.0 (Chicago, IL, USA) and the significance level was set at p = 0.05. Results are shown as mean spectral power (µV^2^/Hz) ± standard deviation.

## Results

Of the 958 participants genotyped for the *ADA* G22A polymorphism, sleep EEG spectral analysis data were available from a total of 800 individuals. Of these, 450 were female (56.3%), 259 (32.4%) were positive for OSAS, 123 (15.4%) were classified as insomniacs, 38 (4.8%) were positive for RLS, 513 (64.1%) had at least one of the above sleep disturbances and 78 (9.8%) were taking medication that influences sleep. The mean age was 42.13±14.27 years, mean BMI was 26.59±5.27 kg/m^2^, and *ADA* A allele frequency was 4.69%. In addition, *ADA* G22A genotype distributions were according do Hardy-Weinberg Equilibrium (Χ^2^ = 0.966; p = 0.326). Due to the low frequency of AA genotype carriers (N = 3) these individuals were grouped together with GA genotype carriers). No associations between *ADA* G22A polymorphism and sleep disturbances were detected (p>0.05, data not shown).

Spectral data were obtained from all derivations (C3-A2, C4-A1, O1-A2, and O2-A1) and for all sleep stages (1, 2, 3+4, and REM) and compared between carriers of the GG genotype (GG genotype group) and carriers of the A allele (GA+AA genotypes group). In the occipital O2-A1 derivation during sleep stage 1, A allele carriers showed higher spectral power than GG allele carriers in the delta (17.65±2.57 vs. 15.02±2.58, p<0.001), theta (4.67±1.11 vs. 4.32±1.21, p = 0.020), and beta 1 bands (1.05±0.37 vs. 0.95±.38, p = 0.045). The GA+AA group also showed increased power in stage 2 in the theta band (5.72±1.38 vs. 4.16±1.12, p<0.001), in stages 3+4 in the delta band (24.95±2.71 vs. 21.02±2.32, p<0.001), and in REM sleep in the theta band (4.40±1.22 vs. 4.02±1.13, p = 0.007). In addition, they showed higher spectral power in the theta band in C3-A2 during stage 2 (5.16±1.21 vs. 3.13±0.79, p<0.001) and in C4-A1 during REM sleep (4.25±1.11 vs. 3.74±1.00, p<0.001). Table 1 summarizes the statistically significant results of the spectral analysis and the Table S1 shows the results for all comparisons.

**Table 1:** Statistically significant differences regarding electroencephalogram (EEG) spectral power values between *ADA* G22A genotype groups in the EPISONO epidemiological sample.

EEG spectral power			*ADA* genotype groups					
Derivation	Bandwidth	Sleep stage	N	GG	N	GA+AA	t	p
O2-A1	Delta	1	670	15.02±2.58	69	17.65±2.57	−8.071	<0.001
O2-A1	Theta	1	670	4.32±1.21	69	4.67±1.11	−2.336	0.020
O2-A1	Beta1	1	670	0.95±0.38	69	1.05±0.37	−2.011	0.045
O2-A1	Theta	2	727	4.16±1.12	72	5.72±1.38	−9.285	<0.001
O2-A1	Delta	3+4	713	21.02±2.32	72	24.95±2.71	−13.486	<0.001
O2-A1	Theta	REM	722	4.02±1.13	71	4.40±1.22	−2.727	0.007
C3-A2	Theta	2	727	3.13±0.79	72	5.16±1.21	−13.984	<0.001
C4-A1	Theta	REM	721	3.74±1.00	71	4.25±1.11	−4.062	<0.001

Results are shown by EEG derivations, bandwidth and sleep stage;

Independent samples t-test with EEG spectral power Z-score transformed value;

Results are shown as mean spectral power ± standard deviation;

Spectral power values are represented in µV^2^/Hz.

**Figure 1:**
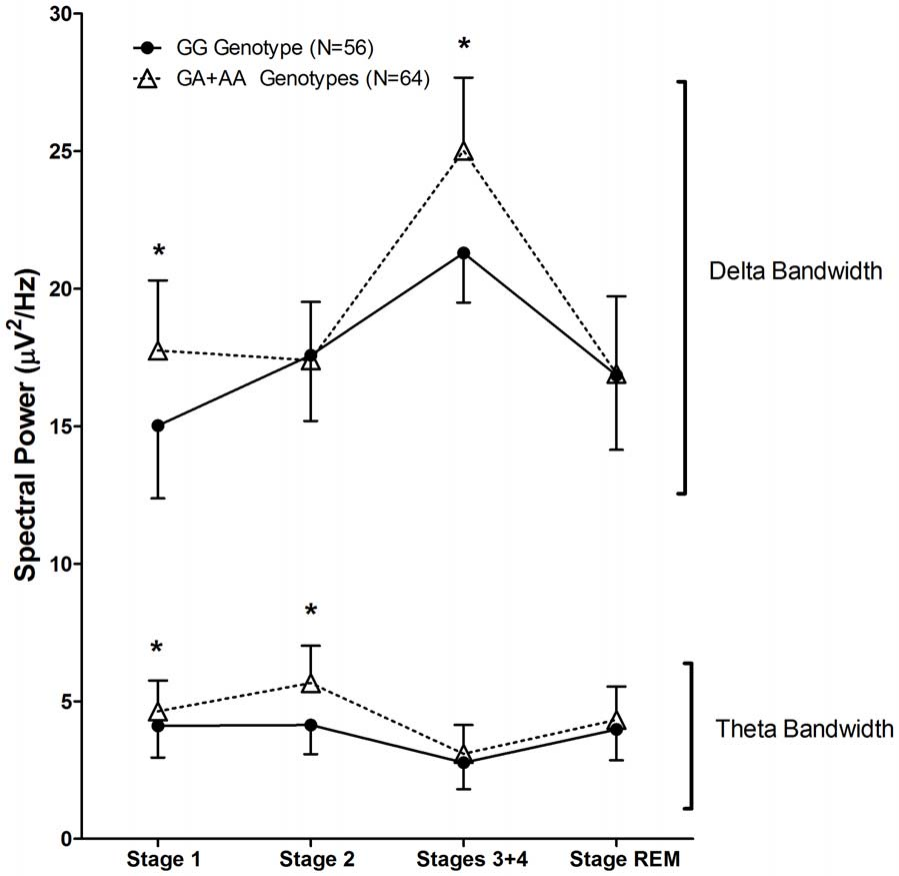
Weighted spectral power means between the *ADA* G22A genotype groups (delta and theta bands) in the matched sample for all sleep stages. Error bars: standard deviation. *p<0.05.

To confirm the aforementioned results, we conducted a more specific analysis using a statistical algorithm to match the GG genotype and A allele carriers for age, gender, BMI, European ancestry, presence of sleep disturbance, use of medication that could influence sleep, and caffeine intake status in the day prior to the polysomnography. The algorithm determined the weight each aforementioned variable is given, found an optimal balance for matching based on a propensity score and selected a subsample for analysis. Fifty-six individuals carrying the GG genotype were matched and compared with 64 carriers of the A allele. To validate the matching algorithm, all the variables were compared between the genotype groups and no differences were found (p>0.05, Table 2). Subsequently, a general linear model using the weights calculated by the matching algorithm was applied to verify the observed effects of the *ADA* polymorphism on spectral power, and to estimate the effect sizes and observed powers for each comparison. Differences in delta spectral power in stages 1 and 3+4 and in theta power in stages 1 and 2 for the O2-A1 derivation remained significant. The differences found for the C3-A2 and C4-A1 were still significant (Table 3). These results confirmed that the *ADA* polymorphism influenced sleep EEG spectral power as observed in the whole sample. As a final point, with the purpose to visually detect changes across all sleep stages, Figure 1 shows a comparison of the spectral power in the delta and theta ranges between the genotype groups.

**Table 2:** General characteristics of the subsample matched using *MatchIt*.

	*ADA* genotype groups		
Variable	GG (N = 56)	GA+AA (N = 64)	p
Age (years)	41.38±14.03	41.77±14.63	0.882^a^
Female gender	38 (58.9%)	39 (60.9%)	0.823^b^
Body mass index (kg/m^2^)	25.98±4.78	25.80±5.28	0.848^a^
Consumed caffeine^c^	38 (67.9%)	43 (67.2%)	0.938^b^
Presence of Sleep disturbance^d^	37 (66.1%)	42 (65.6%)	0.802^b^
Use of Sleep-disturbing medication^e^	6 (10.7%)	7 (10.9%)	0.969^b^
European Ancestry (%)	79.52±15.07	80.20±14.69	0.802^a^

a Independent sample t-test;

b Chi-square test;

c Report of at least one cup of caffeine-containing drinks (coffee, tea, soft drinks or energy drinks) in the day prior to the polysomnography;

d Obstructive Sleep Apnea Syndrome, Restless Legs Syndrome or DSM-IV Insomnia;

e Phytotherapics, antidepressants, benzodiazepines, anticonvulsants, muscle relaxants, or zolpidem.

**Table 3:** Weighted electroencephalogram (EEG) spectral power means between *ADA* G22A genotype groups in the matched sample.

EEG spectral power			*ADA* genotype groups					
Derivation	Bandwidth	Sleep stage	GG (N = 56)	GA+AA (N = 64)	F	p	η^2^	Obs power
O2-A1	Delta	1	15.02±2.64	17.75±2.56	32.873	<0.001	21.8%	100.0%
O2-A1	Theta	1	4.11±1.51	4.63±1.12	1.038	0.013	5.1%	70.6%
O2-A1	Beta1	1	0.92±0.37	1.04±0.38	3.248	0.074	2.7%	43.2%
O2-A1	Theta	2	4.14±1.06	5.67±1.36	45.817	<0.001	28.0%	100.0%
O2-A1	Delta	3+4	21.30±1.80	25.02±2.66	77.827	<0.001	39.7%	100.0%
O2-A1	Theta	REM	3.97±1.21	4.32±1.22	2.587	0.110	2.1%	35.8%
C3-A2	Theta	2	3.22±0.75	5.08±1.17	103.747	<0.001	46.8%	100.0%
C4-A1	Theta	REM	3.62±0.94	4.18±1.10	8.399	0.004	6.6%	82.0%

Estimates of the effect size (η^2^) and observed power are also shown;

Weighted general linear model of EEG spectral power in matched *ADA* genotype groups individuals;

Individuals are matched in regard to age, sex, body mass index, caffeine intake status, European ancestry, sleep disturbance (Obstructive Sleep Apnea Syndrome, Restless Legs Syndrome or DSM-IV Insomnia) and sleep-disturbing medication (phytotherapics, antidepressants, benzodiazepines, anticonvulsants, muscle relaxants, or zolpidem);

Results are shown as weighted mean spectral power ± standard deviation;

Spectral power values are represented in µV^2^/Hz.

## Discussion

This research is a secondary analysis of a previous study that investigated the effect of the *ADA* G22A polymorphism on sleep parameters measured by polysomnography [12]. That study showed that individuals carrying the A allele who consumed caffeine in the day prior to polysomnography demonstrated higher sleep efficiency and a greater REM sleep percentage, after correction for age, gender, and European ancestry. The present findings complement the previous results and comprehensively characterize the effect of the *ADA* G22A polymorphism on sleep structure, in a representative population from São Paulo.

Slow wave oscillations in the EEG during sleep may reflect the homeostatic regulation of both sleep need and intensity. The lower ADA activity in carriers of the A allele might be associated with higher homeostatic load in sleep regulatory areas of the brain, and this may be reflected in the EEG variations observed. We found increased spectral power in A allele carriers in the delta, theta, and beta 1 bands during NREM sleep and in the theta band during REM sleep. A recent study showed a positive correlation between EEG delta power and adenosine levels in the basal forebrain and frontal cortex of rats, after different sleep deprivation periods [8]. These findings give support to the hypothesis that individuals carrying the less active variant of ADA have lower adenosine clearance and, consequently, more slow wave activity and a higher sleep homeostatic load. In addition, we have also demonstrated that carriers of the A allele have higher spectral power in frequency bands other than delta, such as beta 1 in NREM sleep, and theta in both REM and NREM sleep. Corroborating our findings, Bachmann *et al*. have previously described that the differences in the *ADA* G22A genotypes could be extended to theta and alpha oscillations, and were present also in REM sleep [14].

Retéy et al. and Bachmann et al. described for the first time the effects of the *ADA* G22A polymorphism on sleep structure, EEG spectral power, and response to sleep deprivation [13], [14]. Both studies were well-designed and had rigorous experimental protocols, including only healthy individuals that were asked to abstain from caffeine and alcohol, and maintain a regular sleep schedule before the experiments. The present study aimed to replicate the findings of these two studies in an epidemiological sample, and to test whether these associations remained significant in a different population with a larger number of individuals, under no experimental control. Interestingly, the effects of the *ADA* G22A polymorphism on sleep EEG were similar to those found by the controlled studies, suggesting that the findings hold true in different experimental settings and with different sampling approaches. However, as the number of individuals in an epidemiological sample increases, the population tends to become more heterogeneous in a number of socio-demographic, lifestyle, and clinical variables. To address this issue and examine the findings in a more homogeneous sample without disregarding the sources of heterogeneity, we selected GG and GA+AA subjects that were matched for several variables using a statistical algorithm. Associations between the *ADA* G22A polymorphism and changes in delta/theta power in NREM sleep, and theta power in REM sleep, remained significant with high estimates of effect size and observed power. These findings corroborate the results from the whole population and previous findings from other studies [13], [14]. In this sense, it is important to note that, although the prevalence of clinically defined sleep disturbances (OSAS, Insomnia and RLS combined) in the present sample is high (64.1%) and represents the real scenario of the Brazilian population from São Paulo, our analytic strategy using matched individuals was able to account for the potential confounding effect of these disorders and helped to elucidate the real influence exerted by the genetic variant in a population level.

The changes in EEG spectral power across sleep stages give insights about sleep architecture and homeostasis. As shown in Figure 1 for the matched sample, the higher delta spectral power in NREM might be specific for certain sleep stages (1 and 3+4). Likewise, theta spectral power was higher only in REM and stage 2 NREM sleep. Bachmann *et al.* similarly found that slow wave activity was high in the first NREM sleep episode and declined subsequently [14]. Although the present results did not consider each sleep episode independently, taken together, these two studies agree that A allele carriers have deeper sleep and are under higher sleep pressure.

Although we reported relevant findings that contributed to the characterization of the link between genetic variation and sleep homeostasis, the present study has some limitations that deserve to be pointed out. At first, due to the relatively low frequency of *ADA* G22A polymorphism we could not assess the effect of a multiplicative or additive genetic model on sleep EEG spectral power. This approach would be of great interest and is encouraged to be performed in even larger studies with sleep EEG data and *ADA* G22A genotyping. In addition, since we used the Rechtschaffen & Kales criteria we did not recorded frontal lobe data. Once delta activity is thought to be associated to the frontal regions [25], the effect of *ADA* G22A polymorphism on the frontal electrodes would give additional evidence to the reported findings. Another important issue is the absence of standardized waking EEG recordings, once it might improve the relationship between *ADA* G22A polymorphism and sleep homeostasis in humans. More specific studies with high density EEG and animal models are encouraged to assess whether the A allele would have distinct influence on the EEG profile of different brain regions. Nevertheless, our findings could bring opportunity to investigate more specific topics in the field of genetics of sleep EEG profiles.

In conclusion, the present findings suggest that the sleep of individuals carrying the A allele is more intense, as evidenced by higher sleep EEG spectral power in relevant frequency bands. Through its effects on specific components of the sleep EEG, the *ADA* G22A polymorphism may be considered as an important source of variation in sleep homeostasis in humans.

## Supporting Information

Table S1 Electroencephalogram spectral power for all derivations, bandwidths, and sleep stages between *ADA* G22A genotype groups in the EPISONO epidemiological sample. Independent samples t-tests were performed and the whole sample was included.
(XLS)
